# Non-Invasive Tests as a Replacement for Liver Biopsy in the Assessment of MASLD

**DOI:** 10.3390/medicina61040736

**Published:** 2025-04-16

**Authors:** Julia Frączek, Aleksandra Sowa, Paulina Agopsowicz, Maciej Migacz, Katarzyna Dylińska-Kala, Michał Holecki

**Affiliations:** 1 Student Scientific Society at the Department of Internal, Autoimmune and Metabolic Diseases, School of Medicine, Medical University of Silesia, 40-055 Katowice, Poland; s86203@365.sum.edu.pl (A.S.); s85563@365.sum.edu.pl (P.A.); 2 Department of Internal, Autoimmune and Metabolic Diseases, Faculty of Medical Sciences in Katowice, Medical University of Silesia, 40-055 Katowice, Poland; maciek.migacz@gmail.com (M.M.); kasiadylinska@gmail.com (K.D.-K.); holomed@gmail.com (M.H.)

**Keywords:** MASLD, liver disease, FIB-4, liver elastography, liver biopsy

## Abstract

Metabolic dysfunction-associated steatotic fatty liver disease (MASLD) is a worsening global health issue, affecting over one-third of the adult population and representing the leading cause of liver-related morbidity and mortality. MASLD is not only a key precursor to chronic liver disease, but also a systemic condition that leads to numerous extrahepatic complications, increasing the risk of cardiovascular diseases, chronic kidney disease, type 2 diabetes, and certain cancers. The primary reference method for assessing liver fibrosis, allowing for precise determination of its location and severity, remains liver biopsy. However, it is an invasive procedure and involves certain risks. In recent years, the importance of MASLD diagnosis using noninvasive diagnostic methods has been increasing, including serological markers, methods based on multi-omics, and imaging techniques such as liver elastography. This review presents data on the diagnosis and evaluation of this disease that may find application in future clinical practice. The focus is on presenting both currently used and newly identified noninvasive diagnostic methods that open up the prospect of gradually replacing biopsy in the diagnosis of MASLD.

## 1. Introduction

The diagnostic process entails a comprehensive medical history, a thorough physical examination, and a variety of diagnostic tests (a range of diagnostic investigations), including laboratory analyses, imaging studies, endoscopic procedures, and histopathological evaluations, often involving tissue collection techniques such as biopsy [1]. Establishing an accurate diagnosis is an important and highly responsible task, as the selection of appropriate diagnostic modalities influences the entire therapeutic process and its outcomes. In specific clinical cases, the optimal diagnostic approach remains a subject of ongoing debate and research [2].

Metabolic dysfunction-associated steatotic liver disease (MASLD) is the leading cause of chronic liver disease, affecting over 30% of the global adult population, and represents the most prevalent etiology of liver-related morbidity and mortality [3,4]. MASLD encompasses a spectrum of progressive liver steatosis disorders, ranging from isolated hepatic steatosis to metabolic dysfunction-associated steatohepatitis (MASH), accompanied with varying degrees of liver fibrosis, that may ultimately progress to liver cirrhosis [3]. The primary risk factors for MASLD include obesity (particularly visceral obesity), type 2 diabetes, dyslipidemia, and polycystic ovary syndrome [5]. As a systemic disease, it causes complications beyond the liver, leading to cardiovascular diseases, chronic kidney disease, type 2 diabetes, and certain extrahepatic cancers [6,7,8].

In recent years, significant advancements have been made in MASLD research, including updates in nomenclature and the development of non-invasive diagnostic techniques [4].

In 2020, as a result of the increasing prevalence and progress in the knowledge of MASLD pathogenesis, a panel of experts from 22 countries proposed a new definition, MAFLD, to replace the term “non-alcoholic fatty liver disease” (NAFLD) [9]. Subsequently, in June 2023, three major hepatology associations introduced the term MASLD, which achieved broad consensus through an international Delphi panel [10]. The adoption of the term “steatotic liver disease” was intended to eliminate the stigma associated with the word “fatty”, while the revised nomenclature not only enhances understanding of the disease’s pathophysiology by incorporating its underlying metabolic components, but also improves patient classification and enables more accurate risk prediction for both liver-related and cardiovascular complications [10,11].

However, the evolution of MASLD extends beyond nomenclature changes. At every stage of combating the disease, beginning with a diagnosis, various solutions are being explored. Despite being an invasive, costly, and time-consuming procedure with potential complications, liver biopsy remains the gold standard in the exclusionary diagnostics of this disease [12]. Nevertheless, the clinical utility of non-invasive diagnostic modalities has been steadily increasing, including serological markers, methods based on multi-omics, and imaging techniques such as liver elastography [13]. Liver elastography, performed via magnetic resonance (MR) or ultrasound, has gained prominence due to its rapid execution, accuracy, and reproducibility, making it a valuable tool for the quantitative assessment of liver fibrosis [12]. In this review, we will discuss the current status of non-invasive diagnostic tests for MASLD, focusing on the comparison of liver elastography with the gold standard, liver biopsy.

## 2. Diagnosis Process

### 2.1. Criteria for the Diagnosis of MASLD (Metabolic Dysfunction-Associated Steatotic Liver Disease)

**Figure 1:**
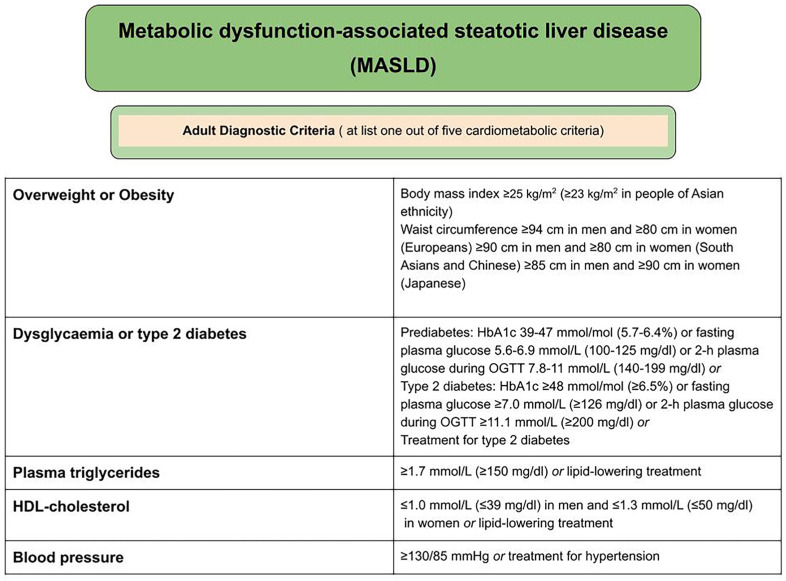
Cardiometabolic determinants in the classification of MASLD [15].

The diagnosis is established in individuals with hepatic steatosis, identified through imaging or liver biopsy, who meet at least one metabolic criterion (Figure 1). Other causes of liver steatosis must be excluded, including medication use, viral hepatitis, pancreatic disorders, inflammatory bowel diseases, and other conditions, with alcohol consumption restricted to less than 20 g/day in women and less than 30 g/day in men [14].

When analyzing patients with MASLD, those most at risk of further fibrosis progression are patients with MASH (metabolic-associated steatohepatitis) [15]. It is characterized by the simultaneous occurrence of hepatocyte ballooning, with or without fibrosis, steatosis, and cellular inflammation. If MASH is in the “at-risk” stage, it may lead to cirrhosis and hepatocellular carcinoma [14,15]. Therefore, according to experts, the ongoing monitoring of patients with MASLD in this respect is extremely important. Patients meeting the above-mentioned criteria for the diagnosis of MASLD should be assessed for the risk of advanced liver fibrosis [14]. According to AASLD, people who abuse alcohol or have a positive family history of liver cirrhosis are at increased risk. First-degree relatives of individuals with NASH-related cirrhosis are 12 times more likely to develop advanced fibrosis. Therefore, targeted screening tests are also recommended for these groups [16].

Current guidelines for the diagnosis, assessment of severity, and treatment of MASLD are widely known and available. They have been developed by many leading organizations dealing with liver diseases, including the American Association for the Study of Liver Diseases (AASLD), the European Association for the Study of the Liver (EASL), the American Association of Clinical Endocrinology (AACE), and the American Diabetes Association (ADA) [14,15,16,17,18].

### 2.2. Serum Markers

**Figure 2:**
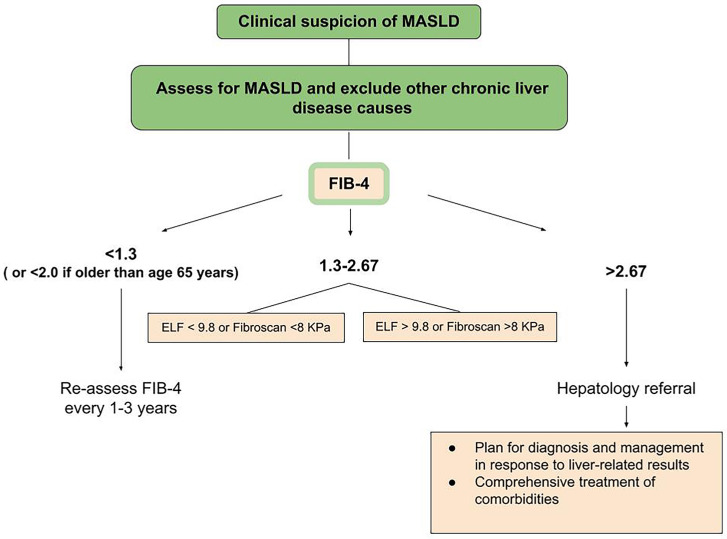
Suggested strategy for non-invasive evaluation of the likelihood of advanced fibrosis and liver-related complications in individuals with metabolic risk factors or symptoms of SLD [18].

Of the many biochemical markers that have been developed, including Fibrosis Score-4 (FIB-4), AST to Platelet Ratio Index (APRI), and NAFLD Fibrosis Score (NFS), the recommended initial non-invasive test is FIB-4 [14]. This index is calculated based on an algorithm that uses the patient’s age, ALT and AST levels, and platelet count. The justification for its use is low cost and ease of implementation [14,16]. FIB-4 has proven prognostic properties in monitoring changes in liver fibrosis over time [19]. Therefore, it is considered the most validated among the indicators used for this purpose. However, it should be remembered that FIB-4 results are influenced by age, making the index less reliable in patients under 35 or over 65 years of age [15]. For patients with a FIB-4 score > 1.3, AGA and AASLD suggest using a combination of at least two non-invasive tests (NITs), combining serum biomarkers and/or imaging-based biomarkers, rather than a single NIT. Both organizations also mention the possibility of using a serum biomarker such as ELF [14,15] (Figure 2).

In one prospective study, the effectiveness of three biochemical markers was evaluated. The NFS scales were compared with ELF (increased liver fibrosis test) and FIB-4 with respect to liver elastography as the standard. The ELF assay outperformed the other two metrics in terms of false positive rates—11% compared to 35% for FIB-4 and 45% for NFS. At the same time, it maintained a low false negative rate of less than 8% [20].

Interestingly, in patients suspected of liver diseases, European guidelines recommend calculating a simple score using platelet count, ALT, and AST [21]. However, in many patients with diagnosed MASLD, these results may be normal and poorly correlated with the severity of the disease [14].

Due to the fact that a very large proportion of MASLD patients are patients with type II diabetes, it is worth noting that, in these patients, AST and ALT activity levels are typically normal, with only GGTP activity being significantly elevated. Moreover, the commonly used NFS scale should be avoided in patients with type 2 diabetes and obesity, as it may overestimate the degree of advanced fibrosis [17,22,23].

PCOS (polycystic ovary syndrome) is another independent risk factor for MASLD and its complications, with available meta-analyses and population studies showing a significant (2–4-fold) increase in the prevalence of MASLD among individuals with PCOS [15,24,25,26,27]. Therefore, appropriate diagnostic tools are being sought to assess liver condition in relevant groups of patients with this condition [28]. For this purpose, screening tests including abdominal ultrasound to assess potential liver steatosis and evaluation of serum aminotransferase activity are recommended [29]. The AACE and AASLD recommend testing serum ALT levels in adult patients with PCOS [17]. The results of one article indicate the usefulness of the FIB-4 index, shear wave elastography, and serum periostin levels in diagnosing liver fibrosis among women with PCOS [30]. Interestingly, in another study involving young patients with this condition, the chance of developing liver fibrosis, assessed using the FIB-4 index, was low regardless of the PCOS phenotype. Therefore, the need for further research on non-invasive diagnostic methods for MASLD, especially among young patients, including those with PCOS, was emphasized [28].

The literature contains numerous studies highlighting the role of individual serological markers in differentiating MASLD [13]. One of the latest retrospective studies involving 100 participants examined the serum marker Fetuitin-A. At a cutoff value of >702.5, this marker demonstrated high efficacy in predicting MASLD, achieving a sensitivity of 82%, specificity of 90%, and overall accuracy of 86%. Additionally, a significant correlation with ultrasound evaluation and FibroScan using controlled attenuation parameters confirmed the relationship between Fetuin-A levels and the severity of the disease [31].

Due to the fact that dyslipidemia is a risk factor for MASLD, some analyses have examined the influence of serum lipid levels on the increased risk of developing this disease. It has been shown that as the ratio of non-HDL cholesterol to HDL cholesterol (non-HDL-c/HDL-c) increases, the prevalence of MASH gradually rises. Moreover, the same high ratio was found to be a predictor of elevated liver enzyme levels in MASH, indicating its potential role as a marker for MASLD [32]. A recent study involving 44 patients highlighted the usefulness of the LDL-TG/LDL-C ratio in distinguishing MASH from MAFL [33].

One of the most widely studied biomarkers in this field is cytokeratin-18 (CK-18), which is a key intermediate filament protein in hepatocytes and a marker of apoptosis in these cells [34]. Multiple studies have indicated a positive correlation between the level of CK-18 and the occurrence of MASH, at the same time confirming its high sensitivity and specificity in the diagnosis of this stage of MASLD [34,35,36].

An advantage over the use of single biomarkers are panels that combine multiple indicators simultaneously, as highlighted by recent studies [13]. Lars Verschuren et al. presented a set of three markers closely associated with the mechanism of collagen turnover and involved in the regulation and progression of liver fibrosis. The proposed panel may be useful in evaluating patients with MASLD and early-stage or severe liver fibrosis. Moreover, its accuracy surpassed the FIB-4 index across all performance metrics for stages F2 and F3/F4 of liver fibrosis [37]. Taking into account that MASH can progress to liver cirrhosis without visible symptoms, preliminary differentiation between MASH and MASL helps in the risk assessment of MASLD-affected patients. With this awareness, Xiang Zhang et al. proposed a group of three parameters (adjusted body mass index [BMI], C-X-C motif chemokine ligand 10 [CXCL10], and cytokeratin 18 fragments M30 [CK-18]) that allowed patients with MASH to be identified among those with MASLD, achieving 90.0% specificity. The indicated panel, N3-MASH, was proven to differentiate patients in this context more effectively compared to single serum biomarkers, and its diagnostic accuracy was validated in three independent cohorts [38].

Non-invasive tests (NITs) are a valuable tool in the diagnosis of MASLD; however, their interpretation should take certain limitations into account. Currently available NITs may exhibit variability and limited accuracy, and their results can be influenced by factors such as PCOS, obesity, or type 2 diabetes [15,17,18]. Moreover, while they allow for the assessment of disease severity, they do not yet enable a definitive diagnosis of MASH or a comprehensive evaluation of the antifibrotic efficacy of new therapeutic approaches. Therefore, their use should be part of a comprehensive clinical assessment tailored to the individual characteristics of each patient [13,14].

### 2.3. Elastography

When fatty liver disease is suspected, ultrasound imaging is typically the initial diagnostic approach. However, conventional ultrasound (CUS) has certain limitations: high interobserver variability and difficulty in detecting mild steatosis in MASLD [29]. Nevertheless, liver biopsy, the gold standard for diagnosing fibrosis, is associated with the risk of complications and variability of results. For this reason, transient elastography (TE), recommended by the WHO, has emerged as a widely adopted alternative method to assess liver stiffness as a biomarker of fibrosis, with high diagnostic accuracy [39].

Elastography techniques are broadly classified into two main categories: ultrasound (US)-based elastography and magnetic resonance imaging (MRI)-based elastography. The first group is further subdivided into qualitative techniques, such as strain elastography and real-time elastography, and quantitative techniques, which include vibration-controlled transient elastography (VCTE), 2D shear wave elastography (2D-SWE), and point shear wave elastography (pSWE) [40]. Among these, VCTE is one of the most widely used methods in elastography. Liver fibrosis is evaluated using the liver stiffness measurement (LSM) provided by VCTE. More recently, VCTE’s capabilities have been expanded with the introduction of the controlled attenuation parameter (CAP), which measures the ultrasonic attenuation of echoes and enables the quantification of hepatic steatosis. As hepatic fat accumulation and inflammation increase, the CAP value progressively rises, demonstrating high accuracy in detecting severe hepatic steatosis [14,40,41].

The American Gastroenterological Association (AGA) and the American Association for the Study of Liver Diseases (AASLD) have recently developed clinical care pathways to support the screening and management of MASLD patients. In both pathways, liver stiffness measurements (LSMs) derived from VCTE are used to assess the risk of advanced fibrosis [14,42]. The AASLD noted that changes in liver stiffness may also be useful not only in diagnosis, but also in detecting disease progression; on VCTE or MRE, a 20% increase in liver stiffness may indicate disease progression [42]. This is of particular importance, because finding noninvasive methods to track changes in disease severity is currently one of the most important needs in the clinical management of MASLD [43]. According to ADA, transient elastography (LSM) is the best-validated imaging technique for fibrosis risk stratification, and it predicts future cirrhosis and all-cause mortality in MASLD [44].

As elastography’s role in clinical practice continues to grow, there is increasing interest in using this tool to assess liver health in patients with conditions such as diabetes, obesity, and cardiovascular diseases. Additionally, its potential role in diagnosing other liver diseases, including bile duct cancer and cirrhosis, is being explored [45]. Recent studies confirm the high prevalence of MASLD, identified by TE, among patients with type 2 diabetes mellitus; thus, there is a high need for systematic screening for MASLD to assess the degree of liver steatosis and fibrosis in this group of patients. Due to its invasive character and unsuitability for large-scale screening, liver biopsy has limited use in patients with type 2 diabetes. Consequently, most screening guidelines for this population prioritize noninvasive markers, such as FIB-4, often combined with TE. This combination enables the effective identification of individuals at both low and high risk of advanced liver fibrosis. Among elastographic methods, TE has already established a position in MASLD diagnosis in patients with type 2 diabetes mellitus [46,47].

Research indicates that patients with PCOS exhibit higher CAP and LSM values compared to controls, although the difference in LSM is not statistically significant. These findings suggest that young women with PCOS may be in the early stages of MASLD development, highlighting an opportunity for early intervention and preventive strategies to slow disease progression and reduce potential complications. Preliminary results from this study indicate that CAP and LSM measurements obtained through elastography could serve as predictive markers for MASLD in women with PCOS [48].

Several epidemiological and genetic studies classify dyslipidemia as a known pathogenic factor of MASLD [49]. Since high plasma triglycerides or low HDL-cholesterol present with abdominal obesity are indications for the preliminary diagnosis of MASLD, there is significant need for non-invasive screening methods, as the early detection of fibrosis and appropriate management can potentially prevent progression to cirrhosis. Imaging techniques such as ultrasound and MR-based elastography can be useful tools for excluding advanced fibrosis as well as predicting the risk of liver-related events and mortality [16].

The integration of FibroScan with additional imaging methods (CT, MRI) has enhanced its capabilities, enabling the monitoring of liver fibrosis progression, including in patients affected by diseases such as COVID-19 [45].

One of the key advantages of elastography is its relatively short examination duration and availability; it can be completed in under 5 min at outpatient imaging centers using standard-of-care ultrasound equipment available from all major vendors on various modern cart-based systems [50]. Moreover, it can be performed at the bedside and the results are immediate. It is a painless procedure with no absolute contraindications or known adverse bioeffects, requiring only a brief breath-hold, typically lasting less than 15 s [50,51]. Ultrasound elastography provides high test repeatability, both when performed by the same operator and by different operators [52]. While performing SWE, a conventional abdominal ultrasound can be carried out during the same visit to assess morphologic signs of cirrhosis, detect portal hypertension, or screen for hepatocellular carcinoma [50]. Similar conclusions were drawn from a prospective study demonstrating that magnetic resonance elastography (MRE) provides excellent reproducibility [53]. The total duration of MRE, including patient positioning and sequence planning, is approximately 10 min. Furthermore, during MRE, T2-weighted coronal and axial sections can be obtained for comparison and to screen other organs (pancreas, gallbladder, spleen), often allowing the detection of incidental lesions that might be overlooked during standard ultrasound imaging [54].

Patients’ perceptions are no less important than the diagnostic outcomes and clinical utility of the elastography. Integrating VCTE into routine clinical practice could significantly reduce the barriers associated with referral-based approaches, the need for multiple visits, and the resulting lack of compliance. VCTE can streamline the diagnostic process and facilitate earlier intervention, potentially improving patient outcomes, particularly among high-risk populations. As the global burden of MASLD continues to grow, the adoption of VCTE in primary care and communities may play a key role in broad-based screening and monitoring efforts [55,56]. For even greater convenience, a new device has been introduced—a palm-sized, wireless elastography system (Liverscan); it offers a convenient alternative to VCTE with comparable performance and very high correlation with LSM results. This new device opens up new possibilities for primary home care in out-of-hospital settings and screening on a larger scale [57,58].

In addition to the development of more compact devices, extensive research is being conducted into advanced artificial intelligence (AI) algorithms for analyzing FibroScan data, enabling the prediction of disease risk, the assessment of liver fibrosis degree, and the prediction of treatment response. The results of meta-analyses highlight the promising potential of AI systems for optimizing MASLD diagnostics [45].

Although transient elastography is widely regarded as an operator-independent technique with very high interobserver agreement, a large review of TE assessments over a five-year period revealed that 3.1% of liver stiffness measurements (LSMs) were unsuccessful and 15.8% were unreliable. Various studies suggest that the main limitation seems to be the use of the M probe in obese patients; it led to higher LSMs and a higher false positive rate. This limitation has been overcome with the introduction of XL probes [40,59,60]. At this point, it is worth mentioning the EUS-SWE technique, which shows promising results for screening and staging liver fibrosis, particularly in obese patients with MASLD, achieving high accuracy across fibrosis thresholds [61]. A recent study examining the effectiveness of Guided-VCTE, an enhanced version of FibroScan, included 130 patients of varying BMIs across multiple centers. The study demonstrated that Guided-VCTE significantly enhances the precision of liver stiffness measurements and reduces the time required for liver localization, particularly in individuals with class II obesity (BMI ≥ 35 kg/m^2^). The newly introduced shear wave propagation prediction index demonstrated a 97% sensitivity in identifying normal liver stiffness measurements. Researchers suggest that Guided-VCTE streamlines and accelerates the FibroScan process, especially for obese patients, potentially expanding its clinical application [62].

Clinicians conducting elastography must be mindful of certain limitations. Conditions such as cholestasis and elevated ALT levels can result in artificially increased LSM values, leading to potentially inaccurate outcomes. Therefore, elastography testing should be delayed until the patient’s condition permits its safe and accurate execution; or, in the case of abnormal ALT levels, it is possible to use ALT-based LSM cutoff values for LSM results interpretation or probability-based LSM results that include ALT [63]. Another limitation is the presence of ascites, which can disrupt shear wave propagation, particularly in the case of TE. According to guidelines, TE is not recommended for patients with perihepatic ascites [63]. The solution may lie in the use of 2D-SWE, a method demonstrated to be more effective than TE. Both techniques are suitable for patients with ascites less than 10 mm or a skin-to-capsule distance of less than 25 mm, as 2D-SWE and TE both exhibit excellent inter-operator agreement and provide reliable measurements that are minimally affected by operator experience [64].

### 2.4. Liver Biopsy

Liver biopsy is an invasive procedure that remains crucial in contemporary clinical practice, providing essential histological insights into liver tissue [65]. Various scoring systems have been developed to assess factors like inflammation and fibrosis, aiding in the diagnosis, prognosis, and management of chronic liver diseases (CLDs), including MASLD. The scoring systems include the Ishak, METAVIR, Scheuer, and Batts–Ludwig scores, with the METAVIR and Ishak systems being the most widely used. The METAVIR score assesses fibrosis on a 5-point scale, where F0 indicates no fibrosis, and F4 corresponds to cirrhosis. Patients with F2 or higher are considered to have “significant fibrosis”, while those with F3 or higher are classified as having advanced fibrosis. The Ishak system uses a 7-point scale, where F0 denotes no fibrosis, F5 indicates incomplete cirrhosis, and F6 represents definite cirrhosis [66,67].

However, indications for liver biopsy have diminished due to the advancement of non-invasive imaging techniques, along with comprehensive patient history, physical examination, and laboratory analysis [68]. Various liver biopsy techniques are available, including percutaneous, transvenous, laparoscopic, and plugged biopsy, with the choice of method depending on patient factors such as bleeding risk. Absolute contraindications for liver biopsy include uncooperative patients, elevated bleeding risk (INR > 1.5 or platelet count < 60,000), and the presence of vascular liver tumors.

Since many patients with liver disease experience abnormal hematological parameters, with disturbances in both thrombolysis and coagulation, traditional measures like platelet count and prothrombin time may not offer reliable guidance for deciding whether to proceed with the procedure. Relative contraindications include ascites and morbid obesity [69]. As an invasive procedure, liver biopsy carries inherent risks, ranging from mild to life-threatening, such as pain, hemorrhage, hypotension, visceral perforation, pneumothorax, and, in rare cases, death [70].

Approximately 60% of complications are identified within 2 h post-procedure, and 96% within 24 h. Fatal complications typically occur within the first 6 h. Hospitalization rates for complications following percutaneous liver biopsy (PLB) range from 1.4% to 3.2%, with pain or hypotension being the most common causes. Mortality rates after liver biopsy have been reported to range from 0.0088% to 0.3% [69].

Percutaneous liver biopsy has more limitations, such as technical challenges. A relatively small portion of the liver is sampled during the procedure, which may lead to underdiagnosis, as liver damage often occurs unevenly across the organ in various diseases. A liver biopsy sample represents roughly 1/50,000th of the liver mass, which may not reflect the entire liver’s condition, particularly in diseases that cause heterogeneous liver damage. Standard practice usually requires that at least six portal triads are present in the biopsy sample to consider it adequate for the diagnostic evaluation of diffuse liver diseases. However, recent findings suggest that these criteria may be insufficient for adequately grading fibrosis and staging inflammation in chronic liver diseases [68] (Table 1).

## 3. Discussion

In response to the increasing prevalence of major risk factors for MASLD, such as obesity, dyslipidemia, and others, the incidence of MASLD and MASH continues to rise, which justifies continuous monitoring and research in this area [14]. Taking into account the diversity of available biomarkers and their limitations, the non-invasive assessment of liver fibrosis in the course of this disease constitutes a significant diagnostic challenge. However, due to their advantages over liver biopsy, these methods are increasingly attracting the attention of researchers and are becoming more widely used. According to the recommendations of the AGA and AASLD, the presented literature emphasized the importance of the FIB-4 index as a screening tool, which has been the most validated among the indicators used for this purpose [13,14,15,16,17,18]. On the other hand, considering studies analyzing various biomarkers, the ELF test has demonstrated a lower rate of false positive results compared to FIB-4 and NFS [20]. These results highlight the need for a multifaceted approach to the diagnosis of MASLD, especially in cases of borderline FIB-4 results, where combining at least two non-invasive tests is recommended [14,15]. The decision to select appropriate diagnostic tools should consider not only their availability and cost, but also the specific characteristics of the studied population, including comorbidities such as type 2 diabetes or PCOS [17,22,23,28]. Conversely, the analysis of new markers, such as fetuin-A or CK-18, shows promising results in differentiating MASLD and its advanced stages [31,34,35]. In particular, biomarker panels combining markers related to collagen turnover and inflammation may represent a future approach to disease diagnosis [37,38].

Ultrasound elastography offers a safe, portable, cost-effective alternative to other imaging techniques, such as MRI or CT scans, which require expensive equipment and/or radiation exposure [71]. The cost-effectiveness of different risk stratification strategies for advanced fibrosis in patients with MASLD has been assessed by multiple economic models, including laboratory studies with transient elastography, transient elastography combined with magnetic resonance elastography, and transient elastography alone. All of these models have demonstrated the superior cost-effectiveness of non-invasive methods compared with liver biopsy [71,72,73,74,75]. Another analysis also confirmed that non-invasive risk stratification methods are cost-effective in identifying high-risk patients and reducing unnecessary referrals to specialists, representing one of the first approaches to assess the cost-effectiveness of elastography in MASLD [72]. The two decades of elastography evolution perfectly reflect the innovations and adaptations in healthcare [45]. Despite certain limitations, such as obesity, ascites, cholestasis effects, and elevated ALT levels, technological advancements—including XL probes, Guided-VCTE, and 2D-SWE—have significantly improved measurement accuracy and expanded its clinical applicability [60,61,62,63,64,71]. The development of elastography has given a completely new direction to the treatment of chronic liver disease, promoting cost-effective, noninvasive methods for determining and tracking fibrosis [45].

Liver biopsy remains the gold standard for diagnosing and staging MASLD, yet it is invasive and carries risks. In recent years, significant progress has been made in developing non-invasive methods for evaluation [76]. It is important to note that the staging and grading systems heavily rely on fibrosis assessment, which is influenced by inflammation. A liver biopsy provides only a single snapshot of the disease at a particular time, failing to account for the chronic relapsing or intermittent inflammatory changes commonly seen in MASLD [67].

## 4. Conclusions

The increasing prevalence of MASLD has driven the development of new non-invasive diagnostic methods, which have proven to be effective and cost-efficient alternatives to liver biopsy, the gold standard. These non-invasive methods, with their safety, good tolerance, low cost, availability, and ease of use, are expected to play an increasingly important role in the management of MASLD, helping to reduce diagnostic uncertainty, improve long-term monitoring, and provide a quantitative biomarker for liver fibrosis. The indicators used for patient risk stratification, newly studied biomarker panels, and advances in ultrasound elastography technology discussed in the above review open up the prospect of gradually replacing biopsy in the diagnosis of MASLD.

## Figures and Tables

**Table 1:** A comparison of liver biopsy and elastography methods in evaluating liver fibrosis and steatosis in MASLD [40,45,50,51,54,57,62,63].

Aspect	Liver Biopsy	Liver Elastography
Invasiveness	Invasive, risk of complications	Non-invasive, safe, no known adverse effects
Diagnostic Value	Gold standard, detailed histology	High accuracy for fibrosis and steatosis assessment
Patient Contraindications	Absolute: bleeding risk, uncooperative patients, vascular tumors; relative: obesity, ascites	TE less effective in patients with ascites or obesity (solved via XL probe, Guided-VCTE, or 2D-SWE)
Procedure Time	Time-consuming, requires monitoring	Quick (under 5–10 min), immediate results
Risks and Complications	Pain, bleeding, hypotension, visceral injury, pneumothorax, rare mortality	Painless, no known adverse bioeffects; minor limitations in obese patients or with ascites
Repeatability	Limited due to risks	Easily repeatable, suitable for monitoring
Screening Use	Not suitable for mass screening	Ideal for large-scale screening (e.g., diabetes, obesity)
Cost & Accessibility	Expensive, hospital-based	Cost-effective, widely available (outpatient/bedside)
Technological Advances	Minimal improvements	Continuous innovation (AI, portable devices, Guided-VCTE)
Sampling Coverage	Small liver fragment, risk of sampling error	Assesses larger liver area, less variability

## References

[1] Davis J.L., Murray J.F., Broaddus V.C., Mason R.J., Ernst J.D., King T.E., Lazarus S.C., Murray J.F., Nadel J.A., Slutsky A.S., Gotway M.B. (2016). History and Physical Examination. Murray and Nadel’s Textbook of Respiratory Medicine.

[2] Kim D.H., Kim S.W., Hwang S.H. (2022). Efficacy of non-invasive diagnostic methods in the diagnosis and screening of oral cancer and precancer. Braz. J. Otorhinolaryngol..

[3] Miao L., Targher G., Byrne C.D., Cao Y.Y., Zheng M.H. (2024). Current status and future trends of the global burden of MASLD. Trends Endocrinol. Metab..

[4] Chan W.K., Chuah K.H., Rajaram R.B., Lim L.L., Ratnasingam J., Vethakkan S.R. (2023). Metabolic Dysfunction-Associated Steatotic Liver Disease (MASLD): A State-of-the-Art Review. J. Obes. Metab. Syndr..

[5] Michalopoulou E., Thymis J., Lampsas S., Pavlidis G., Katogiannis K., Vlachomitros D., Katsanaki E., Kostelli G., Pililis S., Pliouta L. (2025). The Triad of Risk: Linking MASLD, Cardiovascular Disease and Type 2 Diabetes; From Pathophysiology to Treatment. J. Clin. Med..

[6] Targher G., Byrne C.D., Tilg H. (2024). MASLD: A systemic metabolic disorder with cardiovascular and malignant complications. Gut.

[7] Kaya E., Yilmaz Y. (2022). Metabolic-associated Fatty Liver Disease (MAFLD): A Multi-systemic Disease Beyond the Liver. J. Clin. Transl. Hepatol..

[8] Younossi Z.M., Golabi P., Price J.K., Owrangi S., Gundu-Rao N., Satchi R., Paik J.M. (2024). The Global Epidemiology of Nonalcoholic Fatty Liver Disease and Nonalcoholic Steatohepatitis Among Patients with Type 2 Diabetes. Clin. Gastroenterol. Hepatol..

[9] Eslam M., Newsome P.N., Sarin S.K., Anstee Q.M., Targher G., Romero-Gomez M., Zelber-Sagi S., Wai-Sun Wong V., Dufour J.F., Schattenberg J.M. (2020). A new definition for metabolic dysfunction-associated fatty liver disease: An international expert consensus statement. J. Hepatol..

[10] Rinella M.E., Lazarus J.V., Ratziu V., Francque S.M., Sanyal A.J., Kanwal F., Romero D., Abdelmalek M.F., Anstee Q.M., Arab J.P. (2023). NAFLD Nomenclature Consensus Group. A multisociety Delphi consensus statement on new fatty liver disease nomenclature. Hepatology.

[11] Solomon A., Negrea M.O., Cipăian C.R., Boicean A., Mihaila R., Rezi C., Cristinescu B.A., Berghea-Neamtu C.S., Popa M.L., Teodoru M. (2023). Interactions between Metabolic Syndrome, MASLD, and Arterial Stiffening: A Single-Center Cross-Sectional Study. Healthcare.

[12] Ozturk A., Olson M.C., Samir A.E., Venkatesh S.K. (2022). Liver fibrosis assessment: MR and US elastography. Abdom. Radiol..

[13] Wang J.L., Jiang S.W., Hu A.R., Zhou A.W., Hu T., Li H.S., Fan Y., Lin K. (2024). Non-invasive diagnosis of non-alcoholic fatty liver disease: Current status and future perspective. Heliyon.

[14] Rinella M.E., Neuschwander-Tetri B.A., Siddiqui M.S., Abdelmalek M.F., Caldwell S., Barb D., Kleiner D.E., Loomba R. (2023). AASLD Practice Guidance on the Clinical Assessment and Management of Nonalcoholic Fatty Liver Disease. Hepatology.

[15] Wattacheril J.J., Abdelmalek M.F., Lim J.K., Sanyal A.J. (2023). AGA Clinical Practice Update on the Role of Noninvasive Biomarkers in the Evaluation and Management of Nonalcoholic Fatty Liver Disease: Expert Review. Gastroenterology.

[16] Kaylan K.B., Paul S. (2024). NAFLD No More: A Review of Current Guidelines in the Diagnosis and Evaluation of Metabolic Dysfunction-Associated Steatotic Liver Disease (MASLD). Curr. Diab Rep..

[17] Cusi K., Isaacs S., Barb D., Basu R., Caprio S., Garvey W.T., Kashyap S., Mechanick J.I., Mouzaki M., Nadolsky K. (2022). American Association of Clinical Endocrinology Clinical Practice Guideline for the Diagnosis and Management of Nonalcoholic Fatty Liver Disease in Primary Care and Endocrinology Clinical Settings: Co-Sponsored by the American Association for the Study of Liver Diseases (AASLD). Endocr. Pract..

[18] European Association for the Study of the Liver (EASL), European Association for the Study of Diabetes (EASD), European Association for the Study of Obesity (EASO) (2024). EASL-EASD-EASO Clinical Practice Guidelines on the management of metabolic dysfunction-associated steatotic liver disease (MASLD). J. Hepatol..

[19] Kamada Y., Munekage K., Nakahara T., Fujii H., Sawai Y., Doi Y., Hyogo H., Sumida Y., Imai Y., Miyoshi E. (2022). The FIB-4 Index Predicts the Development of Liver-Related Events, Extrahepatic Cancers, and Coronary Vascular Disease in Patients with NAFLD. Nutrients.

[20] Kjaergaard M., Lindvig K.P., Thorhauge K.H., Andersen P., Hansen J.K., Kastrup N., Jensen J.M., Hansen C.D., Johansen S., Israelsen M. (2023). Using the ELF test, FIB-4 and NAFLD fibrosis score to screen the population for liver disease. J. Hepatol..

[21] European Association for the Study of the Liver (2021). EASL Clinical Practice Guidelines on non-invasive tests for evaluation of liver disease severity and prognosis—2021 update. J. Hepatol..

[22] Ciba-Stemplewska A., Gorczyca-Głowacka I. (2023). Practical aspects of diagnosing metabolic dysfunction-associated fatty liver disease. Lekarz POZ.

[23] Drolz A., Wolter S., Wehmeyer M.H., Piecha F., Horvatits T., Schulze Zur Wiesch J., Lohse A.W., Mann O., Kluwe J. (2021). Performance of non-invasive fibrosis scores in non-alcoholic fatty liver disease with and without morbid obesity. Int. J. Obes..

[24] Wu J., Yao X.Y., Shi R.X., Liu S.F., Wang X.Y. (2018). A potential link between polycystic ovary syndrome and non-alcoholic fatty liver disease: An update meta-analysis. Reprod. Health.

[25] Doycheva I., Ehrmann D.A. (2022). Nonalcoholic fatty liver disease and obstructive sleep apnea in women with polycystic ovary syndrome. Fertil. Steril..

[26] Spremović Rađenović S., Pupovac M., Andjić M., Bila J., Srećković S., Gudović A., Dragaš B., Radunović N. (2022). Prevalence, Risk Factors, and Pathophysiology of Nonalcoholic Fatty Liver Disease (NAFLD) in Women with Polycystic Ovary Syndrome (PCOS). Biomedicines.

[27] Shengir M., Chen T., Guadagno E., Ramanakumar A.V., Ghali P., Deschenes M., Wong P., Krishnamurthy S., Sebastiani G. (2021). Non-alcoholic fatty liver disease in premenopausal women with polycystic ovary syndrome: A systematic review and meta-analysis. JGH Open.

[28] Migacz M. (2025). Using non-invasive indicators to screen the PCOS population for liver disease—A single-centre study. Endokrynol. Pol..

[29] Vassilatou E. (2014). Nonalcoholic fatty liver disease and polycystic ovary syndrome. World J. Gastroenterol..

[30] Gürkan E. (2023). Evaluation of Liver Fibrosis in Polycystic Ovary Syndrome by Shear Wave Elastography, FIB-4 Score, and Serum Periostin Levels. Endocrinol. Res. Pract..

[31] Elhoseeny M.M., Abdulaziz B.A., Mohamed M.A. (2024). Fetuin-A: A relevant novel serum biomarker for non-invasive diagnosis of metabolic dysfunction-associated steatotic liver disease (MASLD): A retrospective case-control study. BMC Gastroenterol..

[32] Wang D., Wang L., Wang Z., Chen S., Ni Y., Jiang D. (2018). Higher non-HDL-cholesterol to HDL-cholesterol ratio linked with increased nonalcoholic steatohepatitis. Lipids Health Dis..

[33] Fujii Y., Nouso K., Matsushita H., Kariyama K., Sakurai T., Takahashi Y., Chiba H., Hui S.P., Ito Y., Ohta M. (2020). Low-Density Lipoprotein (LDL)-Triglyceride and Its Ratio to LDL-Cholesterol as Diagnostic Biomarkers for Nonalcoholic Steatohepatitis. J. Appl. Lab. Med..

[34] Zhang X., Li J., Jiang L. (2024). Serum Cytokeratin-18 levels as a prognostic biomarker in advanced liver disease: A comprehensive meta-analysis. Clin. Exp. Med..

[35] Albeltaji A., Al-qatati A., Alzaharna M. (2024). Serum Cytokeratin-18 as a Non-invasive Biomarker and its Association with Biochemical Parameters in the Diagnosis of Non-alcoholic Fatty Liver Disease. Jordan Med. J..

[36] Zeng Y., He H., An Z. (2022). Advance of Serum Biomarkers and Combined Diagnostic Panels in Nonalcoholic Fatty Liver Disease. Dis. Markers.

[37] Verschuren L., Mak A.L., van Koppen A. (2024). Development of a novel non-invasive biomarker panel for hepatic fibrosis in MASLD. Nat. Commun..

[38] Zhang X., Zheng M.-H., Liu D., Lin Y., Song S.J., Chu E.S.-H., Liu D., Singh S., Berman M., Lau H.C.-H. (2023). A blood-based biomarker panel for non-invasive diagnosis of metabolic dysfunction-associated steatohepatitis. Cell Metab..

[39] Huang Z.H., Deng M.Q., Lin Y., Ye C.H., Zheng M.H., Zheng Y.P. (2024). Body posture can modulate liver stiffness measured by transient elastography: A prospective observational study. BMC Gastroenterol..

[40] Taru M.-G., Neamti L., Taru V., Procopciuc L.M., Procopet B., Lupsor-Platon M. (2023). How to Identify Advanced Fibrosis in Adult Patients with Non-Alcoholic Fatty Liver Disease (NAFLD) and Non-Alcoholic Steatohepatitis (NASH) Using Ultrasound Elastography-A Review of the Literature and Proposed Multistep Approach. Diagnostics.

[41] Selvaraj E.A., Mózes F.E., Jayaswal A.N.A., Zafarmand M.H., Vali Y., Lee J.A., Levick C.K., Young L.A.J., Palaniyappan N., Liu C.-H. (2021). Diagnostic accuracy of elastography and magnetic resonance imaging in patients with NAFLD: A systematic review and meta-analysis. J. Hepatol..

[42] Marti-Aguado D., Carot-Sierra J.M., Villalba-Ortiz A., Siddiqi H., Vallejo-Vigo R.M., Lara-Romero C., Martín-Fernández M., Fernández-Patón M., Alfaro-Cervello C., Crespo A. Identification of Candidates for MASLD Treatment with Indeterminate Vibration-Controlled Transient Elastography. Clin. Gastroenterol. Hepatol..

[43] Gidener T., Dierkhising R.A., Mara K.C., Therneau T.M., Venkatesh S.K., Ehman R.L., Yin M., Allen A.M. (2023). Change in serial liver stiffness measurement by magnetic resonance elastography and outcomes in NAFLD. Hepatology.

[44] ElSayed N.A., Aleppo G., Aroda V.R., Bannuru R.R., Brown F.M., Bruemmer D., Collins B.S., Cusi K., Das S.R., Gibbons C.H. (2023). Introduction and Methodology: Standards of Care in Diabetes-2023. Diabetes Care.

[45] Ahmed N., Kumari A., Murty R.S. (2024). FibroScan’s evolution: A critical 20-year review. J. Ultrasound.

[46] Cazac G.-D., Lăcătușu C.-M., Mihai C., Grigorescu E.-D., Onofriescu A., Mihai B.-M. (2022). Ultrasound-Based Hepatic Elastography in Non-Alcoholic Fatty Liver Disease: Focus on Patients with Type 2 Diabetes. Biomedicines.

[47] Powell E.E., Wong V.W., Rinella M. (2021). Non-alcoholic fatty liver disease. Lancet.

[48] Chakraborty S., Ganie M.A., Masoodi I., Jana M., Shalimar, Gupta N., Sofi N.Y. (2020). Fibroscan as a non-invasive predictor of hepatic steatosis in women with polycystic ovary syndrome. Indian. J. Med. Res..

[49] Xuan Y., Zhu M., Xu L., Huangfu S., Li T., Liu C., Zhou D. (2024). Elevated non-HDL-C to HDL-C ratio as a marker for NAFLD and liver fibrosis risk: A cross-sectional analysis. Front. Endocrinol..

[50] Pierce T.T., Samir A.E. (2022). Liver Fibrosis: Point-Ultrasound Elastography Is a Safe, Widely Available, Low-Cost, Noninvasive Biomarker of Liver Fibrosis That Is Suitable for Broad Community Use. AJR Am. J. Roentgenol..

[51] Patel K., Sebastiani G. (2020). Limitations of non-invasive tests for assessment of liver fibrosis. JHEP Rep..

[52] Fraquelli M., Rigamonti C., Casazza G., Conte D., Donato M.F., Ronchi G., Colombo M. (2007). Reproducibility of transient elastography in the evaluation of liver fibrosis in patients with chronic liver disease. Gut.

[53] Wang R., Wang Y., Qiu S., Ma S., Yan F., Yang G.-Z., Li R., Feng Y. (2024). A Comparative Study of Three Systems for Liver Magnetic Resonance Elastography. J. Magn. Reson. Imaging.

[54] Kharat A., Vanpully N.S., Jeeson J.C. (2021). Simplified Guide to MR Elastography in Early Detection of Hepatic Fibrosis with Case Reports: The New Norm in Assessing Liver Health. Indian. J. Radiol. Imaging.

[55] Sarkar Das T., Meng X., Abdallah M., Bilal M., Sarwar R., Shaukat A. (2024). An Assessment of the Feasibility, Patient Acceptance, and Performance of Point-of-Care Transient Elastography for Metabolic-Dysfunction-Associated Steatotic Liver Disease (MASLD): A Systematic Review and Meta-Analysis. Diagnostics.

[56] Srivastava A., Jong S., Gola A., Srivastava A., Jong S., Gola A., Gailer R., Morgan S., Sennett K., Tanwar S. (2019). Cost-comparison analysis of FIB-4, ELF and fibroscan in community pathways for non-alcoholic fatty liver disease. BMC Gastroenterol..

[57] Lai J.C.-T., Liang L.Y., Wong G.L.-H. (2024). Noninvasive tests for liver fibrosis in 2024: Are there different scales for different diseases?. Gastroenterol. Rep..

[58] Huang Z.-H., Wang L.-K., Cai S.-Y., Chen H.-X., Zhou Y., Cheng L.-K., Lin Y.-W., Zheng M.-H., Zheng Y.-P. (2024). Palm-Sized Wireless Transient Elastography System with Real-Time B-Mode Ultrasound Imaging Guidance: Toward Point-of-Care Liver Fibrosis Assessment. Diagnostics.

[59] Ferraioli G., Wong V.W.-S., Castera L., Berzigotti A., Sporea I., Dietrich C.F., Choi B.I., Wilson S.R., Kudo M., Barr R.G. (2018). Liver Ultrasound Elastography: An Update to the World Federation for Ultrasound in Medicine and Biology Guidelines and Recommendations. Ultrasound Med. Biol..

[60] Arieira C., Monteiro S., Xavier S., Dias de Castro F., Magalhães J., Marinho C., Pinto R., Costa W., Pinto Correia J., Cotter J. (2019). Transient elastography: Should XL probe be used in all overweight patients?. Scand. J. Gastroenterol..

[61] Wang T.J., Jirapinyo P., Shah R., Schuster K., Papke D.J., Thompson C.C., Doyon L., Lautz D.B., Ryou M. (2025). EUS-guided shear wave elastography for fibrosis screening in patients with obesity and metabolic dysfunction-associated steatotic liver disease: A pilot study (with video). Gastrointest. Endosc..

[62] Bastard C., Audière S., Foucquier J., Lorée H., Miette V., Bronowicki J.-P., Stern C., Caussy C., Sandrin L. (2025). Guided-VCTE: An Enhanced FibroScan Examination With Improved Guidance and Applicability. Ultrasound Med. Biol..

[63] Chang P.E., Goh G.B., Ngu J.H., Tan H.K., Tan C.K. (2016). Clinical applications, limitations and future role of transient elastography in the management of liver disease. World J. Gastrointest. Pharmacol. Ther..

[64] Guo H.Y., Liao M., Zheng J., Huang Z.P., Xie S.D. (2023). Two-dimensional shear wave elastography utilized in patients with ascites: A more reliable method than transient elastography for noninvasively detecting the liver stiffness-an original study with 170 patients. Ann. Transl. Med..

[65] Boyd A., Cain O., Chauhan A., Webb G.J. (2020). Medical liver biopsy: Background, indications, procedure and histopathology. Frontline Gastroenterol..

[66] Sharma S., Khalili K., Nguyen G.C. (2014). Non-invasive diagnosis of advanced fibrosis and cirrhosis. World J. Gastroenterol..

[67] Chowdhury A.B., Mehta K.J. (2023). Liver biopsy for assessment of chronic liver diseases: A synopsis. Clin. Exp. Med..

[68] Reddy K.R. (2024). Liver biopsy: Archaic but resilient and many roads lead to Rome. Clin. Liver Dis..

[69] Chan M., Navarro V.J. (2023). Percutaneous Liver Biopsy. StatPearls.

[70] Aljawad M., Yoshida E.M., Uhanova J., Marotta P., Chandok N. (2013). Percutaneous liver biopsy practice patterns among Canadian hepatologists. Can. J. Gastroenterol..

[71] González-Mateo E., Camarena F., Jiménez N. (2025). Real-time ultrasound shear wave elastography using a local phase gradient. Comput. Methods Programs Biomed..

[72] Congly S.E., Shaheen A.A., Swain M.G. (2021). Modelling the cost effectiveness of non-alcoholic fatty liver disease risk stratification strategies in the community setting. PLoS ONE.

[73] Noureddin M., Jones C., Alkhouri N., Gomez E.V., Dieterich D.T., Rinella M.E., Therapondos G., Girgrah N., Mantry P.S., Sussman N.L. (2021). Screening for Nonalcoholic Fatty Liver Disease in Persons with Type 2 Diabetes in the United States Is Cost-effective: A Comprehensive Cost-Utility Analysis. Gastroenterology.

[74] Crossan C., Majumdar A., Srivastava A., Thorburn D., Rosenberg W., Pinzani M., Longworth L., Tsochatzis E.A. (2019). Referral pathways for patients with NAFLD based on non-invasive fibrosis tests: Diagnostic accuracy and cost analysis. Liver Int..

[75] Johansen P., Howard D., Bishop R., Moreno S.I., Buchholtz K. (2020). Systematic Literature Review and Critical Appraisal of Health Economic Models Used in Cost-Effectiveness Analyses in Non-Alcoholic Steatohepatitis: Potential for Improvements. Pharmacoeconomics.

[76] Hudson D., Afzaal T., Bualbanat H., AlRamdan R., Howarth N., Parthasarathy P., AlDarwish A., Stephenson E., Almahanna Y., Hussain M. (2024). Modernizing metabolic dysfunction-associated steatotic liver disease diagnostics: The progressive shift from liver biopsy to noninvasive techniques. Therap. Adv. Gastroenterol..

